# Impact of 17-alpha ethinyl estradiol (EE2) and diethyl phthalate (DEP) exposure on microRNAs expression and their target genes in differentiated SH-SY5Y cells

**DOI:** 10.1038/s41598-025-86911-1

**Published:** 2025-01-21

**Authors:** Agnese Graziosi, Camilla Corrieri, Giulia Sita, Luca Ghelli, Sabrina Angelini, Roberta d’Emmanuele di Villa Bianca, Emma Mitidieri, Raffaella Sorrentino, Patrizia Hrelia, Fabiana Morroni

**Affiliations:** 1 Department of Pharmacy and BioTechnology – FaBiT, Alma Mater Studiorum – University of Bologna, via Irnerio 48, Bologna, 40126 Italy; 2 Department of Pharmacy, School of Medicine and Surgery, University of Study of Naples – Federico II, via Montesano 49, Naples, 80131 Italy

**Keywords:** Mechanisms of disease, Epigenetics in the nervous system, Environmental impact

## Abstract

Environmental endocrine disruptor chemicals (EDCs) have raised significant concerns due to their potential adverse effects on human health, particularly on the central nervous system (CNS). This study provides a comparative analysis of the effects of 17-alpha ethinyl estradiol (EE2) and diethyl phthalate (DEP) on neuronal cell proliferation and neurotoxicity. Using differentiated SH-SY5Y human neuronal cells, we evaluated cell viability, microRNA (miRNA) regulation, and RNA expression following exposure to subtoxic concentrations of EE2 and DEP. Our results show that both EDCs downregulated specific miRNAs—miR-18b-5p, miR-200a-3p, and miR-653-5p—affecting key processes such as cell proliferation, survival, and apoptosis. Gene expression analysis revealed the upregulation of EGFR, IGF1R, BTG2, and SH3BP4, implicating these miRNAs in the regulation of the Ras and PI3K/Akt/mTOR pathways. Our findings highlight distinct cellular responses: DEP disrupts PTEN activity, while EE2 enhances phosphorylation within the PI3K/Akt/mTOR pathway, promoting pro-survival and anti-apoptotic signals. This study emphasizes the urgent need for regulatory measures to mitigate the neurotoxic effects of EDCs and offers valuable insights into their molecular impacts on brain health.

**Supplementary Information:**

The online version contains supplementary material available at 10.1038/s41598-025-86911-1.

## Introduction

In recent years, concerns have escalated regarding the potential adverse effects of environmental contaminants on human health^1^. According to the U.S. Environmental Protection Agency (EPA), endocrine disruptor chemicals (EDCs) include primarily human-made chemical agents capable of disrupting the synthesis, secretion, transport, binding, or elimination of natural hormones, which are pivotal for maintaining homeostasis, regulating reproduction, fostering development, and influencing behavior^2,3^. Estrogenic EDCs have been extensively studied, but debate persists about the extent to which most EDCs can mimic or counteract estrogen’s effects on receptor transcriptional activities. Consequently, their potential to disrupt various signaling pathways and cellular functions remains a matter of contention^4,5^. Among EDCs, 17-alpha-ethinyl estradiol (EE2) stands out due to its potent estrogenic activity and widespread use in pharmaceutical applications, particularly in oral contraceptives and hormone replacement therapies. EE2 is a synthetic derivative of the natural hormone estradiol, engineered to be orally active and resistant to metabolic degradation^6^. While its pharmacological benefits are well-documented, there is growing concern about its environmental impact and potential health risks associated with its pervasive presence in ecosystems. Upon entering the environment, EE2 has been detected in surface waters, sediments, and even drinking water sources, primarily due to insufficient removal during wastewater treatment processes^7^.

Another class of widely used industrial chemicals known for their estrogenic endocrine-disrupting properties are phthalates, synthetic compounds utilized to enhance the flexibility, transparency, durability, and longevity of plastics. Exposure to phthalates through oral, dermal, and inhalation routes has been linked to developmental and reproductive abnormalities, including sperm damage, early puberty onset, infertility, changes in the reproductive tract, and adverse pregnancy outcomes^8^. Within the phthalate family, exposure to diethyl phthalate (DEP) is more common for humans, being primarily associated with the use of personal care products such as perfumes, shampoo, cosmetics, and detergents^9–11^. Exposure to DEP has been associated with a two-fold increase in the risk of breast cancer^12^, prompting questions about the potential detrimental effect of DEP at non-toxic doses which can occur through a hormonal disruption mechanism. Despite in vivo and in vitro evidence indicating an estrogenic nature of DEP, the mechanisms behind its estrogen-disrupting effects remain controversial^11^.

To date, research efforts have primarily focused on unfolding the mechanisms through which DEP exert its effects on cellular function and it has been associated with endocrine disruption, mitochondrial dysfunction, and epigenetic modifications, all of which can perturb normal cellular homeostasis^13^.

Recent studies have begun to elucidate the impact of DEP exposure on neuronal proliferation and microRNA (miRNA) regulation. DEP has been shown to alter the expression profiles of specific miRNAs involved in cell cycle regulation, apoptosis, and neuronal differentiation, thereby perturbing normal neurodevelopmental processes^14^. Moreover, DEP-induced changes in miRNA expression may contribute to neurotoxicity by disrupting the balance between pro-survival and pro-apoptotic pathways in neuronal cells^15^.

Recently, there has been growing concern regarding the potential neurotoxic effects of EDCs on the central nervous system (CNS). Neuronal cells represent a fundamental component of the CNS, playing pivotal roles in cognitive functions, sensory perception, and motor coordination. Neuronal cell proliferation is a crucial process underlying the development, maintenance, and plasticity of the nervous system^16^. Disruptions in this delicate balance can have deep implications for neurological function and contribute to the pathogenesis of various neurodevelopmental and neurodegenerative disorders. Consequently, any disruption in their proliferation dynamics can have profound implications. Notably, while previous studies have explored the individual impacts of EDCs on various physiological systems, a comprehensive understanding of their effects on cell proliferation deregulation remains conspicuously understudied^17^. Emerging evidence suggests that exposure to environmental pollutants, may interfere with the finely tuned mechanisms governing neuronal cell proliferation, thereby impacting brain health and potentially predisposing individuals to neurodevelopmental disorders and neurodegenerative conditions^18^.

In the present study, by employing a multifaceted approach, we elucidated the differential impacts of EE2 and DEP on key cellular processes, underlying the mechanisms by which these EDCs disrupt normal cell proliferation dynamics in neuronal cells. Understanding the intricate interplay between EDCs exposure, miRNA dysregulation, and proliferation deregulation in neuronal cells is crucial for assessing the neurotoxic potential of EDCs and developing targeted interventions, and regulatory measures to mitigate its adverse effects on brain health. Given the pervasive nature of EE2 and DEP exposure and the potential for significant health impacts, it is crucial to deepen our understanding of their modes of action and to identify effective strategies for mitigating their risks.

## Results

### DEP and EE2 do not significantly affect the viability of differentiated SH-SY5Y cells

**Fig. 1:**
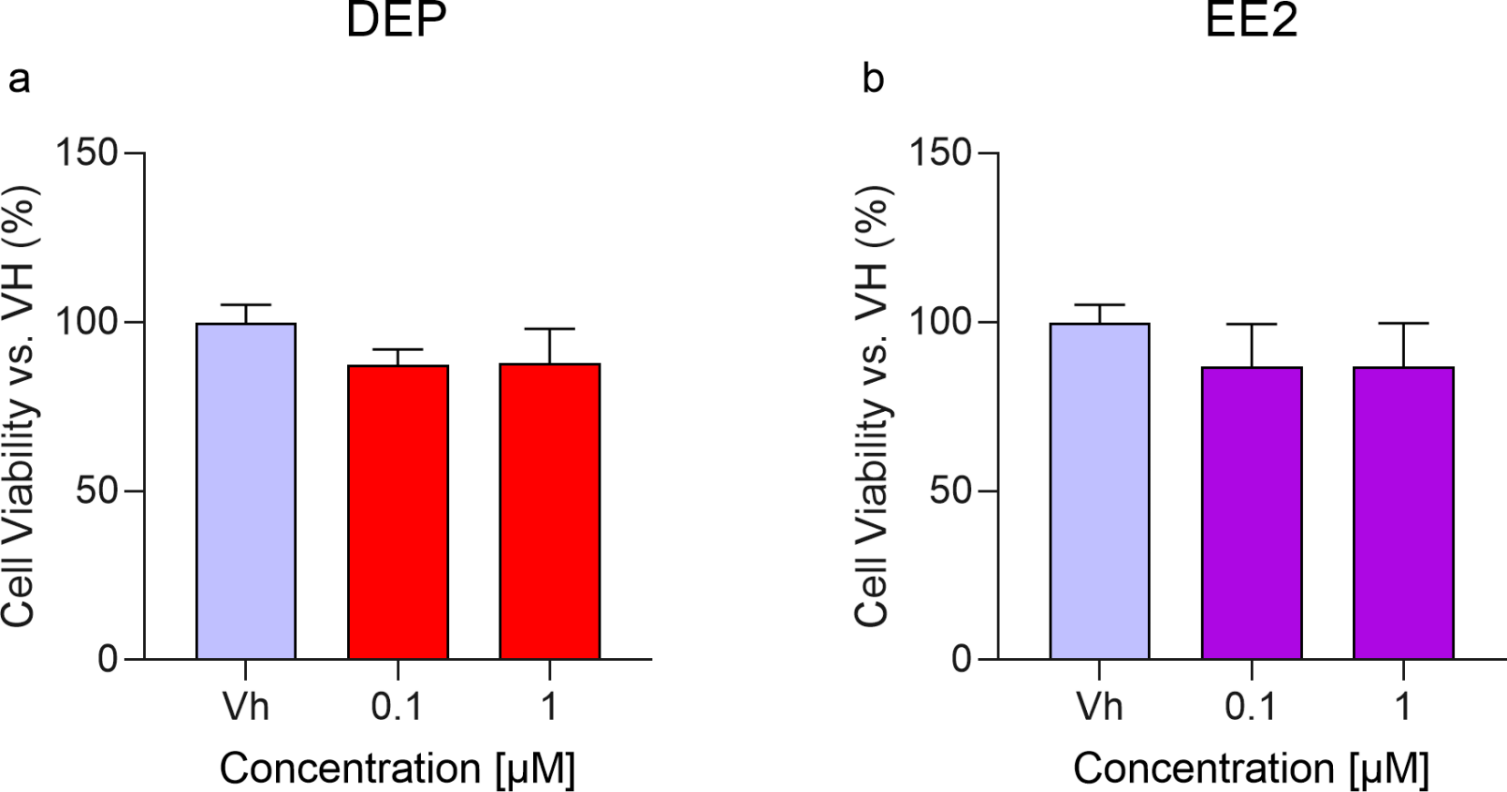
Neuronal viability in differentiated SH-SY5Y after the treatment with DEP (**a**) and EE2 (**b**). Cells were differentiated with retinoic acid and treated for 48 h with EDCs 0.1 or 1 µM. Cell viability was evaluated using the AlamarBlue HSTM test by the conversion of resazurin to resorufin. Data are expressed as the mean ± SD of viability percentage relative to cells treated with the Vh of three independent experiments (One-way ANOVA, post hoc test Dunnet).

AlamarBlue HSTM tests were conducted to assess the potential cytotoxic effects of DEP and EE2^19^. SH-SY5Y cells were induced to differentiate into mature neurons and then treated with EDCs 0.1 and 1 µM for 48 h. The percentage of viability indicated that the EDCs were not cytotoxic to differentiated SH-SY5Y cells compared to the Vh control (Fig. 1).

### Exposure to DEP and EE2 at subtoxic concentrations induces changes in miRNA profile

**Fig. 2:**
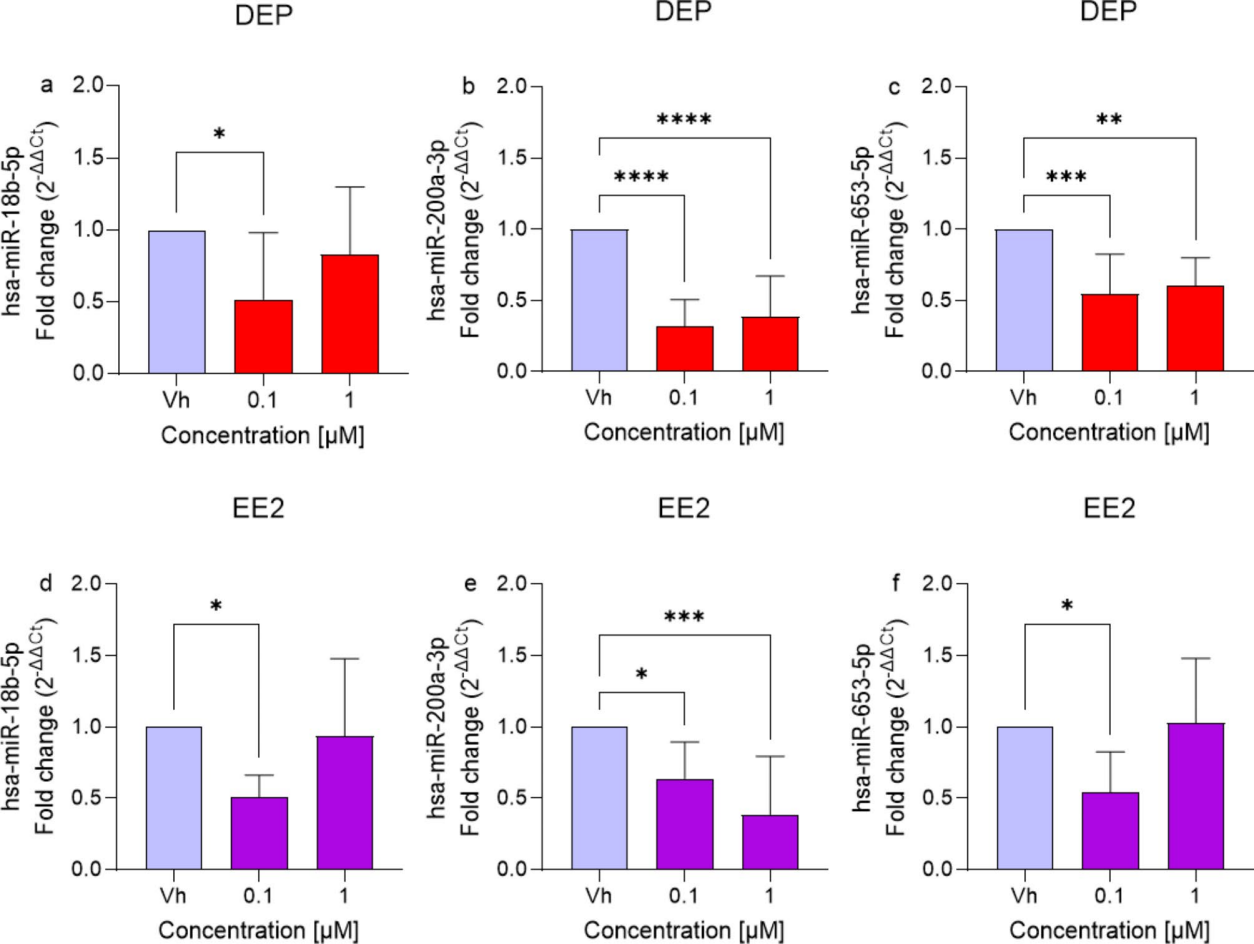
Differential expression of miR-18b-5p, miR-200a-3p, and miR-653-5p in differentiated SH-SY5Y cells treated for 48 h with DEP or EE2 [0.1, 1 µM] by qRT-PCR. Quantitative analysis was performed by the 2^−ΔΔCt^ method and Vh samples were considered as the calibrator of the experiment. Data are expressed as fold increases and reported as mean ± SD of three independent experiments ((**a**,**d**,**f**) *p < 0.05 vs. Vh; (**b**) ****p < 0.0001 vs. Vh; (**c**) **p < 0.01 and ***p < 0.001 vs. Vh; (**e**) *p < 0.05 and ***p < 0.001 vs. Vh; One-way ANOVA, post hoc test Dunnet).

The candidate miRNAs were selected according to their reported or predicted role in neuronal proliferation and survival^20–22^. Among them, miR-18b-5p, miR-200a-3p, and miR-653-5p were selected for qRT-PCR validation in the differentiated SH-SY5Y cells treated with DEP or EE2 [0.1 and 1 µΜ] for 48 h. The expression levels of selected miRNAs were significantly downregulated by EDCs treatment as compared to the Vh (Fig. 2). Specifically, the lowest concentration of both DEP and EE2 reduced the expression of miR-18b-5p (Fig. 2a and d). miR-200a-3p was downregulated at all concentrations of the EDCs tested (Fig. 2b and e). Additionally, miR-653-5p was significantly downregulated by both concentrations of DEP (Fig. 2c) and by the lowest concentration of EE2 (0.1 µM) (Fig. 2f).

### In silico predictions indicate the involvement of biological processes related to neurotoxicity and cell survival

**Fig. 3:**
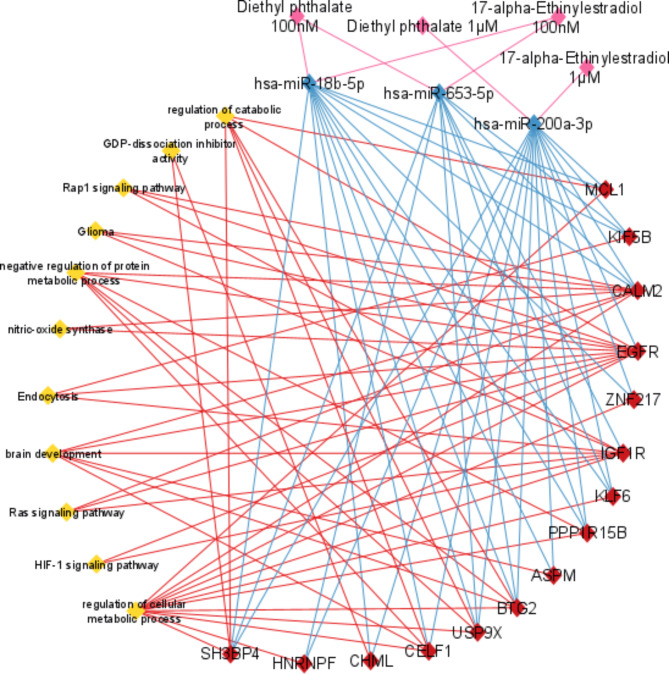
Network created by Cytoscape software that integrates the identified genes, the miRNAs targeting them, their interactions, and the pathways in which they are involved. MiR-18b-5p, miR-200a-3p and miR-653-5p (blue diamonds) likely target various genes (red diamonds) within the identified pathway (yellow diamonds), potentially acting as key regulators in the cellular response to EDCs exposure.

To prioritize high-confidence miRNA-target interactions, we used miRWalk to identify genes predicted as targets by three algorithms: miRWalk, TargetScan, and miRDB. This approach minimizes the number of false positives typically associated with single-tool predictions. Functional enrichment analysis of the prioritized target genes was performed using g: Profiler with the Gene Ontology: Biological Process database to identify pathways potentially regulated by the differentially expressed miRNAs. Finally, Cytoscape software was used to create a network visualization depicting the interactions between miRNAs, their target genes, and the enriched pathways (Fig. 3).

Among the dysregulated pathways it is possible to identify “Ras signaling pathway”. Ras activity modulation, potentially due to miRNA targeting, could be a central driver of neurotoxicity. Uncontrolled Ras activation can lead to increased cell death, disrupting survival pathways and promoting neuronal death. Additionally, it can also interfere with brain development by hindering the proper differentiation and maturation of neuronal cells^23^. Rap 1, another small G protein like Ras, is related to EGFR and IGFR activity, both of which are targets of miRNAs modulated by EE2 and DEP. Rap1 could contribute to neurotoxicity by affecting cell adhesion and migration, processes crucial for forming proper neuronal networks. Imbalances in Rap1 signaling might also influence cell survival pathways, potentially leading to neuronal loss. Indeed, Rap1 and Ras can interact, and dysregulation in one might indirectly affect the other, amplifying the neurotoxic effects. The identified miRNAs may also target components that regulate GDP dissociation from RAS, as shown in the network, potentially affecting its activation state in cells exposed to EDCs^24^. Another pathway “brain development” includes various genes, such as CALM2, EGFR, IGF1R, ASPM, BTG2, CELF1, which could contribute to neurotoxicity. The altered expression of these genes, which are crucial for differentiation, could prevent cells from maturing into functional neurons^25^. Activation of HIF-1 signaling, involving EGFR and IGF1R, might reflect a response to EDCs-induced stress. Chronic activation of this pathway could lead to oxidative stress, damaging cellular components, disrupting neuronal function, and impairing essential neuronal processes^26^.

#### Effects of downregulation of miR-18b-5p, miR-200a-3p and miR-653-5p on EGFR, IGF1R, BTG2 and SH3BP4 gene expression

**Fig. 4:**
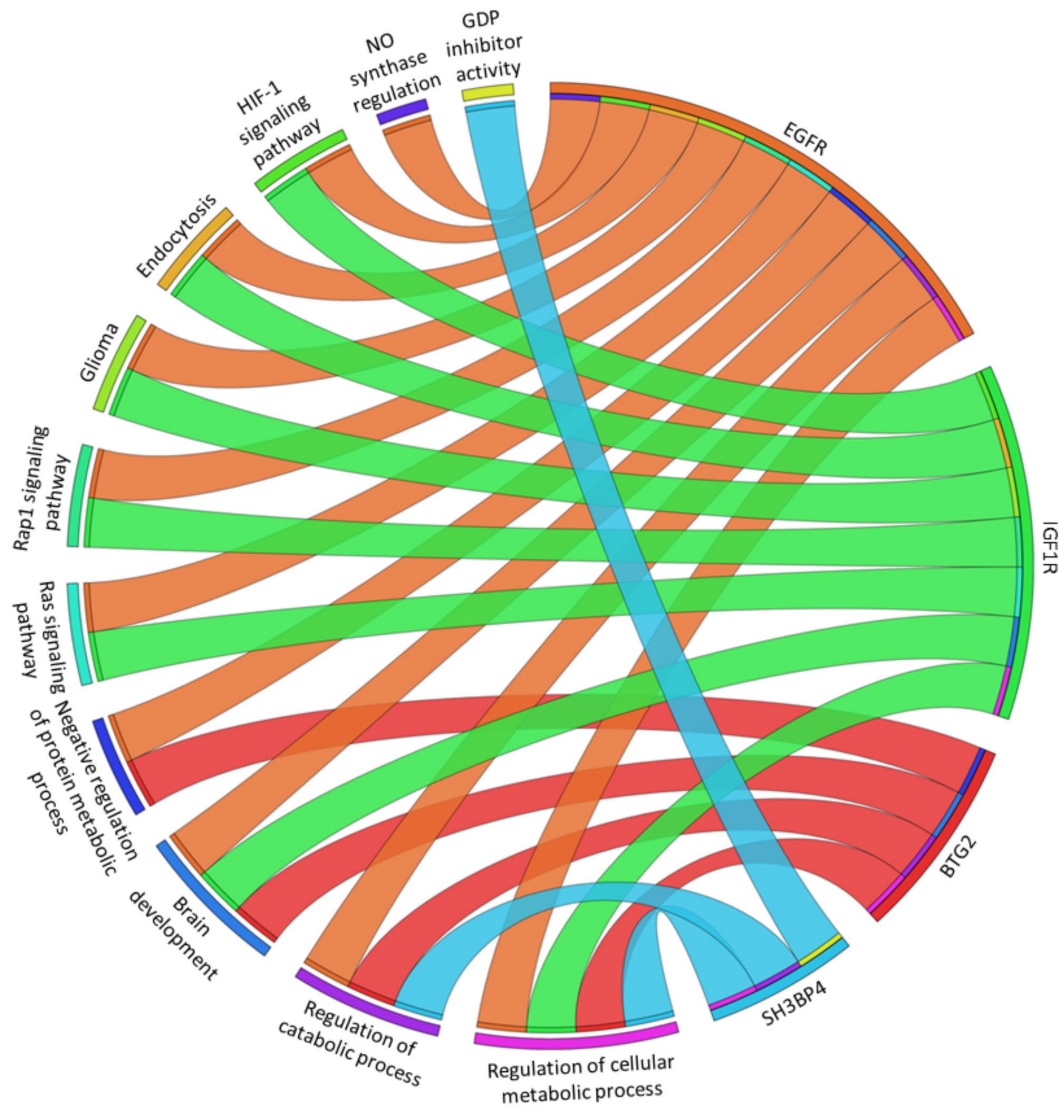
Circular genome data visualization (circos) plot for the selected overrepresented Gene Ontology (GO) terms and corresponding miRNAs target genes. Outside the circle, 11 significantly enriched pathways (on the left) and 4 target genes (on the right) are indicated. Each gene is denoted by a unique color band and the undirected colored edge inside the circle represents the association of a particular gene with its respective pathway(s).

The pathways most implicated in neurotoxicity include Ras signaling pathway, brain development, HIF-1 signaling pathway, glioma development, and GDP dissociation inhibitor. Figure 4 highlights EGFR, IGF1R, BTG2, and SH3BP4 among the identified genes described in Fig. 3. These genes, which are targets of the studied miRNAs, are involved in these pathways.

**Fig. 5:**
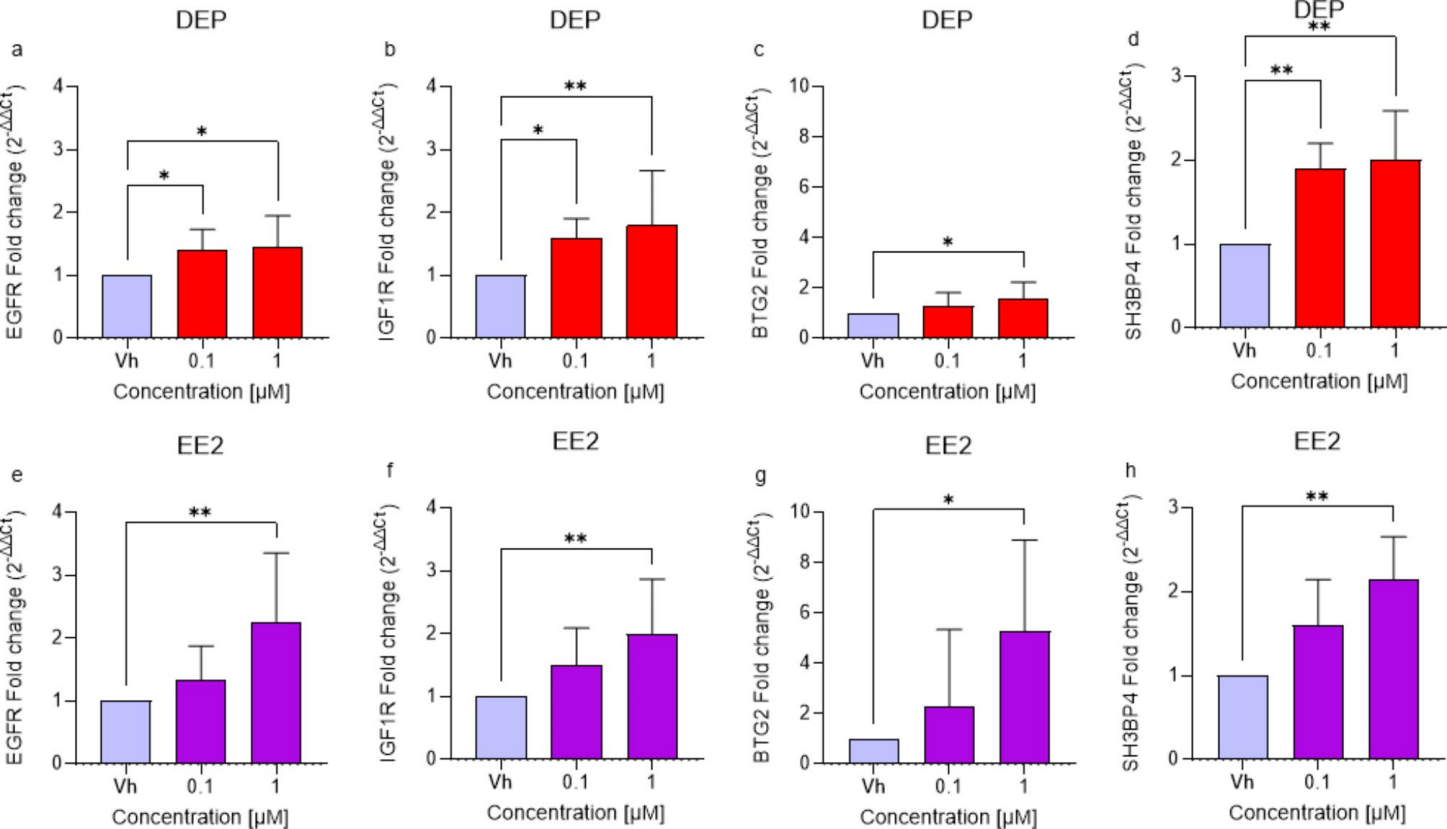
Differential expression of EGFR, IGF1R, BTG2, and SH3BP4 genes in differentiated SH-SY5Y cells treated for 48 h with DEP or EE2 [0.1, 1 µM] by single primer qRT-PCR. Quantitative analysis was performed by the 2^−ΔΔCt^ method and Vh samples were considered as the calibrator of the experiment. Data are expressed as fold increases and reported as mean ± SD of three independent experiments ((**a**,**c**,**g**) *p < 0.05 vs. Vh; (**b**) *p < 0.05 and **p < 0.01 vs. Vh; (**d**–**f**,**h**) **p < 0.01 vs. Vh; One-way ANOVA, post hoc test Dunnet).

Consequently, the gene expression of these targets was investigated individually using single primers (Fig. 5)^27^. The analysis revealed the upregulation of EGFR, IGF1R, and SH3BP4 after the treatment with both concentrations of DEP (Fig. 5a and b, and d), as well as with the higher concentration of EE2 (Fig. 5e and f, and h). Additionally, BTG2 was upregulated following treatment with the higher concentration of both EDCs (Fig. 5c and g).

#### Subtoxic concentrations of DEP and EE2 downregulate miR-200a-3p, miR-18b-5p and miR-653-5p contributing to the activation of EGFR/Ras/p53 pathway in differentiated SH-SY5Y cells

**Fig. 6:**
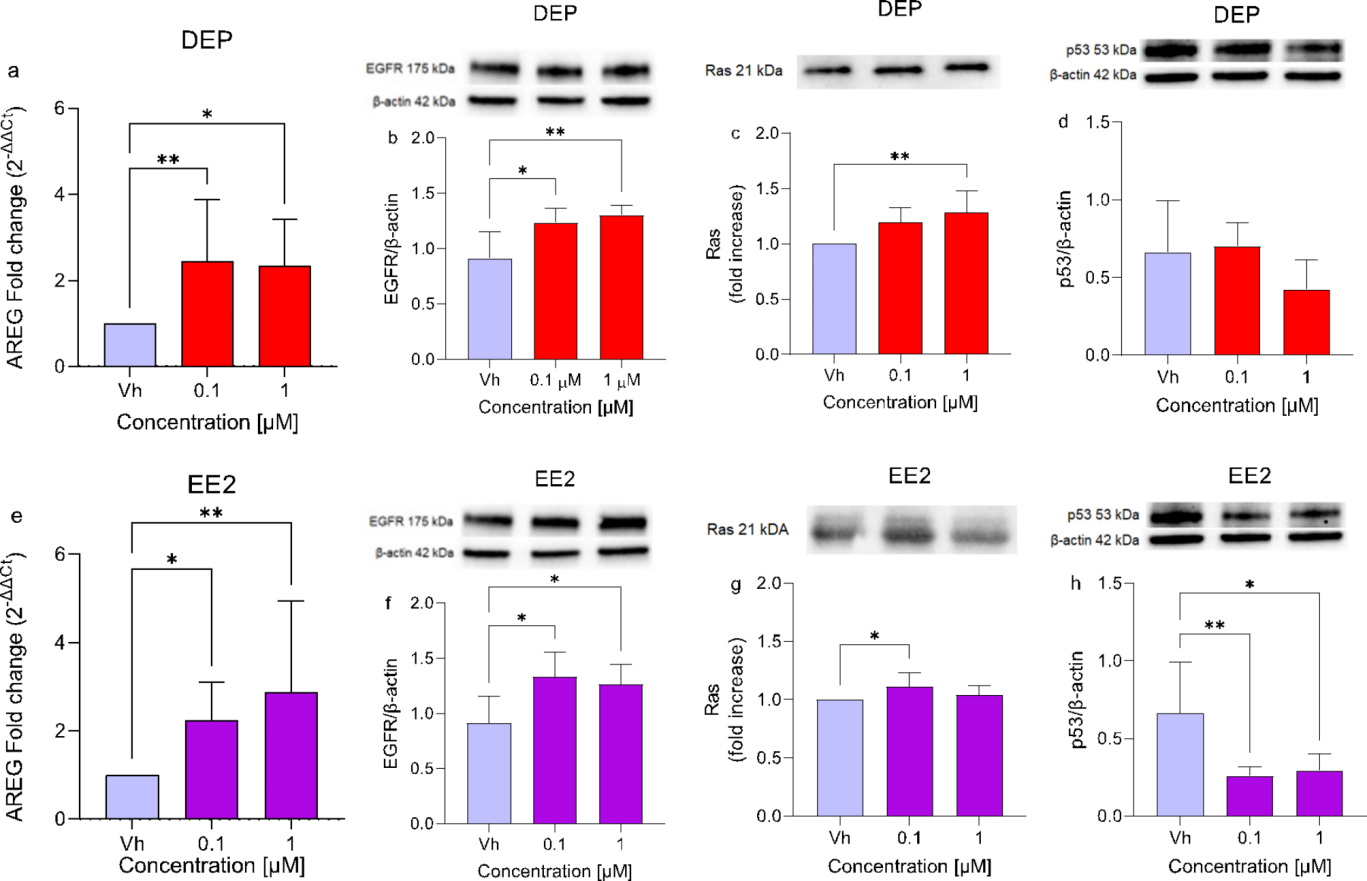
Differential expression of AREG, EGFR, Ras, and p53 in differentiated SH-SY5Y cells treated for 48 h with DEP or EE2 [0.1, 1 µM]. AREG expression (**a**,**e**) was evaluated by single primer qRT-PCR. Quantitative analysis was performed by the 2^−ΔΔCt^ method and Vh samples were considered as the calibrator of the experiment. The proteins levels of EGFR (**b**,**f**), Ras (**c**,**g**), and p53 (**d**,**h**) were determined by Western Blotting at 175, 21, and 53 kDa respectively and using β-actin (42 kDa) as loading control. Top: cropped representative images of the protein of interest expressions. The raw pictures are showed in the supplementary material Bottom: quantitative analysis of the Western blotting results. The graphs show densitometry analysis of the bands appertaining to the protein of interest. Data are expressed as the ratio between the protein of interest and β-actin and reported as mean ± SD of three independent experiments ((**a**,**b**,**e**,**h**) *p < 0.05 and **p < 0.01 vs. Vh; (**c**) **p < 0.01 vs. Vh; (**f**,**g**) *p < 0.05 vs. Vh. One-way ANOVA, post hoc test Dunnett).

Given that EGFR is a target of miR-200a-3p, the EGFR/Ras/p53 signaling pathway was investigated to understand the functional implications of this interaction (Fig. 6). The EGFR family plays a fundamental role in cell proliferation, survival, invasion, and immune evasion^28^. The activation of EGFR, in turn, initiates Ras to inhibit p53 activity, failing in its tumor suppression role^29^. The results showed a significant increase in AREG, a member of the EGF family, and EGFR protein levels after treatment with both EDCs at 0.1 and 1 µM, compared to Vh. (Figure 6a and d). Furthermore, treatment with DEP [1 µM] and EE2 [0.1 µM] resulted in significantly increased levels of Ras (Fig. 6b and e). In contrast, p53 activity was significantly downregulated only with EE2 treatment (Fig. 6c and f).

#### Subtoxic concentrations of DEP and EE2 exert a dual effect on PI3K/Akt/mTOR pathway in differentiated SH-SY5Y cells

**Fig. 7:**
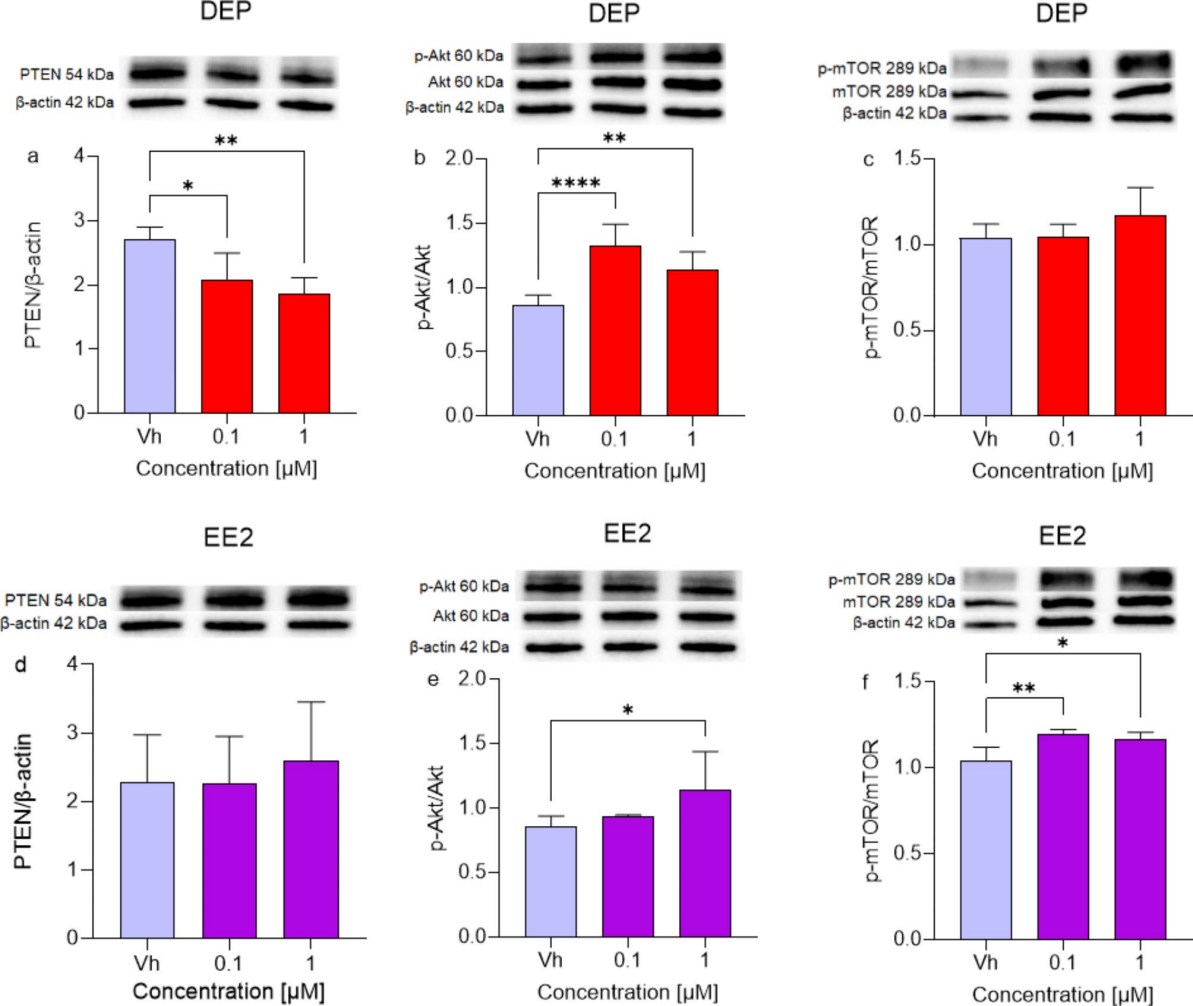
Differential expression of PTEN, and phosphorylation of Akt and mTOR in differentiated SH-SY5Y cells treated for 48 h with DEP or EE2 [0.1, 1 µM]. The proteins levels of PTEN (**a**,**d**), the phosphorylation of Akt (**b**,**e**), and mTOR (**c**,**f**) were determined by Western Blotting at 54, 60, and 289 kDa respectively and using total Akt, mTOR, or β-actin (42 kDa) as loading control. Top: cropped representative images of the protein of interest expressions. The raw pictures are showed in the supplementary material. Bottom: quantitative analysis of the Western blotting results. The graphs show densitometry analysis of the bands appertaining to the protein of interest. Data are expressed as the ratio between the protein of interest and the corresponding loading control and reported as mean ± SD of three independent experiments ((**a**,**f**) *p < 0.05 and **p < 0.01 vs. Vh; (**b**) **p < 0.01 and ****p < 0.0001 vs. Vh; (**e**) *p < 0.05 vs. Vh. One-way ANOVA, post hoc test Dunnett).

PI3K activation may occur via Ras by increased expression of growth factor receptors, such as EGFR or by loss of PTEN. PIP3, the most important second messenger of the PI3K pathway, mediates receptor tyrosine kinase signaling to Akt, which is crucial for cell proliferation and survival^30^. Treatment with DEP [0.1, 1 µM] significantly disrupts PTEN activity (Fig. 7a), leading to increased Akt phosphorylation (Fig. 7b), although this does not result in enhanced mTOR phosphorylation (Fig. 7c). In contrast, EE2 does not impact PTEN activity (Fig. 7d) but significantly enhances the phosphorylation of both Akt and mTOR (Fig. 7e-f).

The increased levels of phosphorylated Akt and mTOR suggest that activation of the PI3K/Akt/mTOR pathway may be driven by upstream signals from EGFR and Ras. Conversely, DEP treatment leads to PTEN downregulation, which can enhance activation of the PI3K/Akt pathway^31^. Despite the increased activation of Akt, the phosphorylation status of mTOR remains unchanged. This indicates that DEP’s effects may be more pronounced upstream of mTOR or that other regulatory mechanisms are keeping mTOR activity at baseline levels.

### Subtoxic concentrations of DEP and EE2 inhibit apoptotic cellular death in differentiated SH-SY5Y cells

Abnormal activation of the PI3K/Akt pathway enhances anti-apoptotic genes expression, such as Bcl-2, and reduces pro-apoptotic genes, such as Bax^32^.

**Fig. 8:**
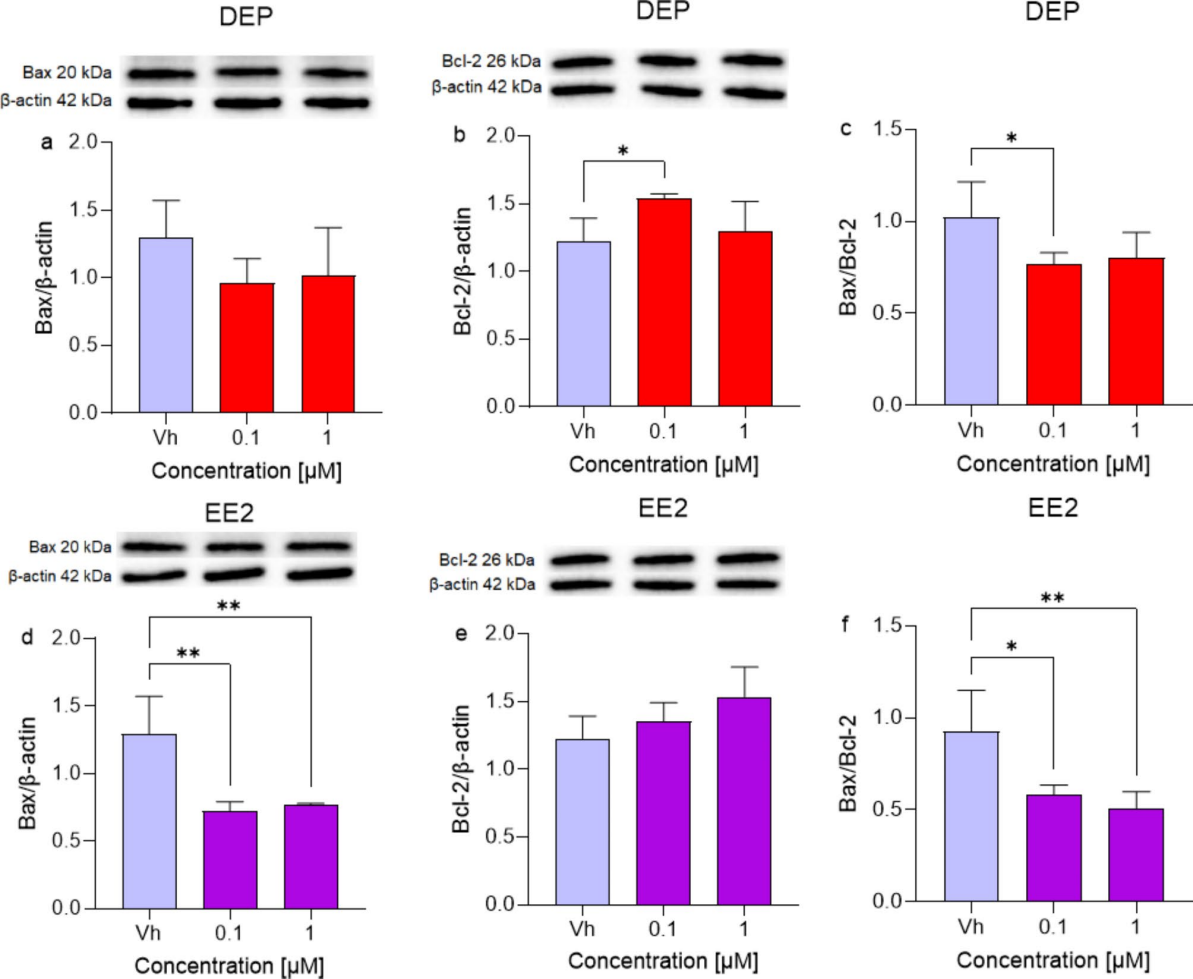
Differential expression of Bax and Bcl-2 in differentiated SH-SY5Y cells treated for 48 h with DEP or EE2 [0.1, 1 µM]. The proteins levels of Bax (**a**,**d**) and Bcl-2 (**b**,**e**) were determined by Western Blotting at 20 and 26, 60 kDa respectively and using β-actin (42 kDa) as loading control. The ratio between Bax and Bcl-2 is represented in panel c (DEP) and in panel f (EE2) Top: cropped representative images of the protein of interest expressions. The raw pictures are showed in the supplementary material. Bottom: quantitative analysis of the Western blotting results. The graphs show densitometry analysis of the bands appertaining to the protein of interest. Data are expressed as the ratio between the protein of interest and β-actin expression and reported as mean ± SD of three independent experiments ((**b**,**c**) *p < 0.05 vs. Vh; (**d**) **p < 0.01 vs. Vh; (**f**) *p < 0.05 and **p < 0.01 vs. Vh. One-way ANOVA, post hoc test Dunnett).

In our experimental conditions, Bax protein levels decreased with both DEP and EE2, though the reduction was significant only with EE2 [0.1, 1 µM] (Fig. 8a, d). Conversely, Bcl-2 levels significantly increased only with the lower concentration of DEP (Fig. 8b), while no significant changes were observed with either concentration of EE2 (Fig. 8e). These results suggest that DEP and EE2 promote cell survival through distinct mechanisms. After DEP treatment, the deregulation of apoptosis appears to be linked to Akt activation, as evidenced by a significant reduction in the Bax/Bcl-2 ratio, favoring cell survival, particularly at the lower concentration (Fig. 8c). In contrast, EE2 treatment reduces p53 levels, which in turn affects Bax expression and leads to a significant decrease in the Bax/Bcl-2 ratio (Fig. 8f).

## Discussion

The widespread use of EE2 in contraceptive pills and its persistence in the environment due to incomplete metabolism and excretion contribute to its ubiquitous presence in water bodies. EE2 is known to disrupt endocrine function in aquatic organisms, leading to feminization of male fish, impaired reproductive success, and altered behavior^33^. Furthermore, EE2 exposure has been linked to the disruption of fish populations in rivers and lakes, with cascading effects on entire aquatic ecosystems^34^. The long-term consequences of EE2 pollution on biodiversity and ecosystem stability underscore the urgent need for stringent regulations and monitoring efforts to mitigate its environmental impact.

Similarly, DEP, a plasticizer commonly used in the production of polyvinyl chloride (PVC) plastics, poses significant risks to environmental pollution. DEP leaches from plastic products into the environment during manufacturing, use, and disposal, contaminating soil, water, and air^35^. As a ubiquitous environmental pollutant, DEP has been detected in various environmental matrices, including surface water, groundwater, sediment, and biota^36^. The widespread distribution of DEP in the environment poses risks to terrestrial and aquatic ecosystems, with potential implications for human health through food chain contamination and exposure.

Here, we elucidated the differential impacts of EE2 and DEP on key cellular processes, underlying the mechanisms by which EE2 and DEP disrupt normal cell proliferation dynamics and homeostasis in neuronal cells. This study demonstrates that while subtoxic concentrations of both EE2 and DEP do not affect cell proliferation, differentiated SH-SY5Y cells respond to the exposure by activating cell survival pathways.

Several studies have shown that exposure to EDCs leads to widespread miRNA alterations, which collectively target pathways involved in cell proliferation, survival, cell cycle regulation, apoptosis, and DNA damage repair^37,38^.

MiR-200a-3p has been extensively studied in the context of neurodegeneration, where it play a role in attenuating amyloid toxicity and oxidative stress in neuronal cells^21^. Similarly, miR-18b-5p has shown potential in neuroprotective therapies due to its role in reducing apoptotic signaling in neurodegenerative models. By modulating calcium pathways, it contributes to neuronal stability and prevents apoptosis triggered by neurotoxic stressors^22^. Also, miR-653-5p has been linked to cell survival pathways in models of oxidative stress injury in neurons. Its regulatory network includes the SIRT1 pathway, which is important for mitochondrial function and neuroprotection^22^. These miRNAs are thus integral to maintaining neuronal health by modulating signaling pathways associated with neuroprotection, apoptosis, and cell survival. On the contrary, in cancer the miRNAs under investigation are strongly associated with tumor progression, metastasis, treatment resistance, and deregulation of oncogenic signaling pathways. Indeed, miR-200a-3p inhibit cancer cell proliferation by inducing apoptosis in a renal carcinoma cellular model^39^. Similarly, as shown by Zhang et al., the overexpression of miR-653-5p in breast cancer inhibits the activation of the Akt/m-TOR pathway and induces apoptosis^40^. The observed effects of DEP and EE2 exposure on differentiated SH-SY5Y cells involve changes in several key signaling pathways and apoptotic regulators, reflecting the compounds’ impact on cellular growth, survival, and apoptosis.

The present study shows a complex and multifaced response of the cells exposed to subtoxic concentrations of EDCs. MiRNAs are versatile regulators that can simultaneously affect multiple signaling pathways. This ability makes them crucial players in maintaining cellular homeostasis and a focus of research for therapeutic interventions in diseases such as cancer. Results presented indicate that miR-200a-3p, miR-18b-5p, and miR-653-5p can interfere simultaneously with both pro-apoptotic and pro-survival pathways. This effect is likely due to the inherent ability of miRNAs to target multiple genes. In 2019, Jin et al. provided evidence that the same miRNAs can be simultaneously involved in both apoptosis-related mechanisms and cell differentiation^41^. Similarly, by targeting genes involved in both pro-apoptotic and anti-apoptotic pathways, a single miRNA can fine-tune the balance between cell survival and death. Indeed, the impact of miRNAs on cellular pathways is often part of a larger regulatory network. MiRNAs can indirectly affect pathways by modulating upstream or downstream components, leading to complex regulatory effects. The specific effects of miRNAs can vary depending on the cellular context, including the presence of other regulatory proteins, the type of cell, and the specific stress or signals the cell is responding to^42^.

In this study, all selected miRNAs deregulate the expression of two genes, SH3BP4 and BTG2, involved in the apoptotic process. BTG2 is a member of the anti-proliferative gene family and plays a critical role in regulating cell growth, differentiation, and apoptosis. The expression of BTG2 interferes with several important cellular processes such as G1/S phases transition and cell proliferation. BTG2 promotes the expression of pro-apoptotic proteins and the inhibition of anti-apoptotic pathways and Akt signaling pathway. Furthermore, BTG2 expression is upregulated by p53. In turn, BTG2 can enhance the stability and activity of p53, creating a positive feedback loop that strengthens the cell’s response to stress and DNA damage^43^. Finally, due to its ability to inhibit cell proliferation, to promote apoptosis and DNA repair, BTG2 functions as a tumor suppressor^44^. Indeed, its downregulation or loss is often observed in various cancers, where it is associated with increased tumor growth and poor prognosis^45^.

Similarly, SH3BP4 is a protein involved in various cellular processes, particularly in the regulation of endocytosis and signaling pathways downstream of EGFR. By regulating the endocytosis and trafficking of EGFR, SH3BP4 affects the intensity and duration of EGFR signaling, which is crucial for cell proliferation and survival^46^.

The role of the PI3K/Akt pathway is known to play a key role in numerous cellular functions including proliferation, adhesion, migration, invasion, metabolism, and survival^47,48^. PI3K activation may occur via Ras, by increased expression of growth factor receptors such as EGFR or by loss of PTEN^49^. Hence, inactivation or loss of PTEN results in constitutively active Akt signaling which promotes cell growth and survival. Following Akt activation, mTOR may be phosphorylated too and the activation of the downstream pathway may be fundamental in controlling cell proliferation and differentiation^50^. In our study, DEP and EE2 treatments upregulate EGFR expression that leads to the activation of Ras and triggers downstream signaling pathways, including the PI3K/Akt/mTOR pathway^51^. Previously also Lee et al. have shown in hepatocellular carcinoma an increased activation of EGFR expression^52^, as well as Fiocchetti et al. have shown that exposure to DEP activates PI3K/Akt signals through ERα activation, promoting proliferation in breast cancer cells^53^.

EE2 and DEP appear to exert their effects by modulating key signaling pathways and proteins that collectively promote cell survival and proliferation while inhibiting apoptosis. The increased EGFR, AREG, and Ras levels suggest activation of growth factor signaling, leading to downstream activation of the PI3K/Akt/mTOR pathway, as evidenced by increased p-Akt and p-mTOR after EE2 treatment. Furthermore, the reduction in PTEN levels contributes to increased Akt phosphorylation, promoting survival and growth signaling. The lack of change in p-mTOR suggests that DEP’s effects might be specific to the upstream components of the pathway or influenced by different regulatory factors. Additionally, the regulation of apoptotic pathways differs between EE2 and DEP exposure. EE2 treatment reduces p53 levels, diminishing apoptotic signals, and selectively decreases Bax, further reducing pro-apoptotic activity without affecting the anti-apoptotic Bcl-2. In contrast, DEP treatment results in increased Bcl-2 levels. Both conditions lead to a decreased Bax/Bcl-2 ratio, enhancing cell survival and apoptosis inhibition.

The cellular response regulated by miRNAs, involving the modulation of both pro-apoptotic and anti-apoptotic pathways, can be considered analogous to hormesis. Hormesis is a dose-response phenomenon characterized by a biphasic response to an agent, where low doses stimulate beneficial effects and high doses induce detrimental effects. MiRNAs can fine-tune the balance between cell survival and apoptosis, much like hormesis maintains a balance between beneficial and harmful effects. For example, miR-200a-3p, miR-653-5p, and miR-18b-5p can target multiple pathways to ensure that cells appropriately respond to various stimuli, avoiding both excessive cell proliferation and unwarranted cell death^40,54–56^. The regulatory functions of miRNAs on apoptotic and survival pathways reflect a hormesis-like phenomenon, where the modulation of gene expression leads to context-dependent and dose-dependent cellular responses. This fine-tuning mechanism ensures cellular homeostasis and adaptive responses to various stimuli, similar to the principles of hormesis observed in toxicology and pharmacology.

**Fig. 9:**
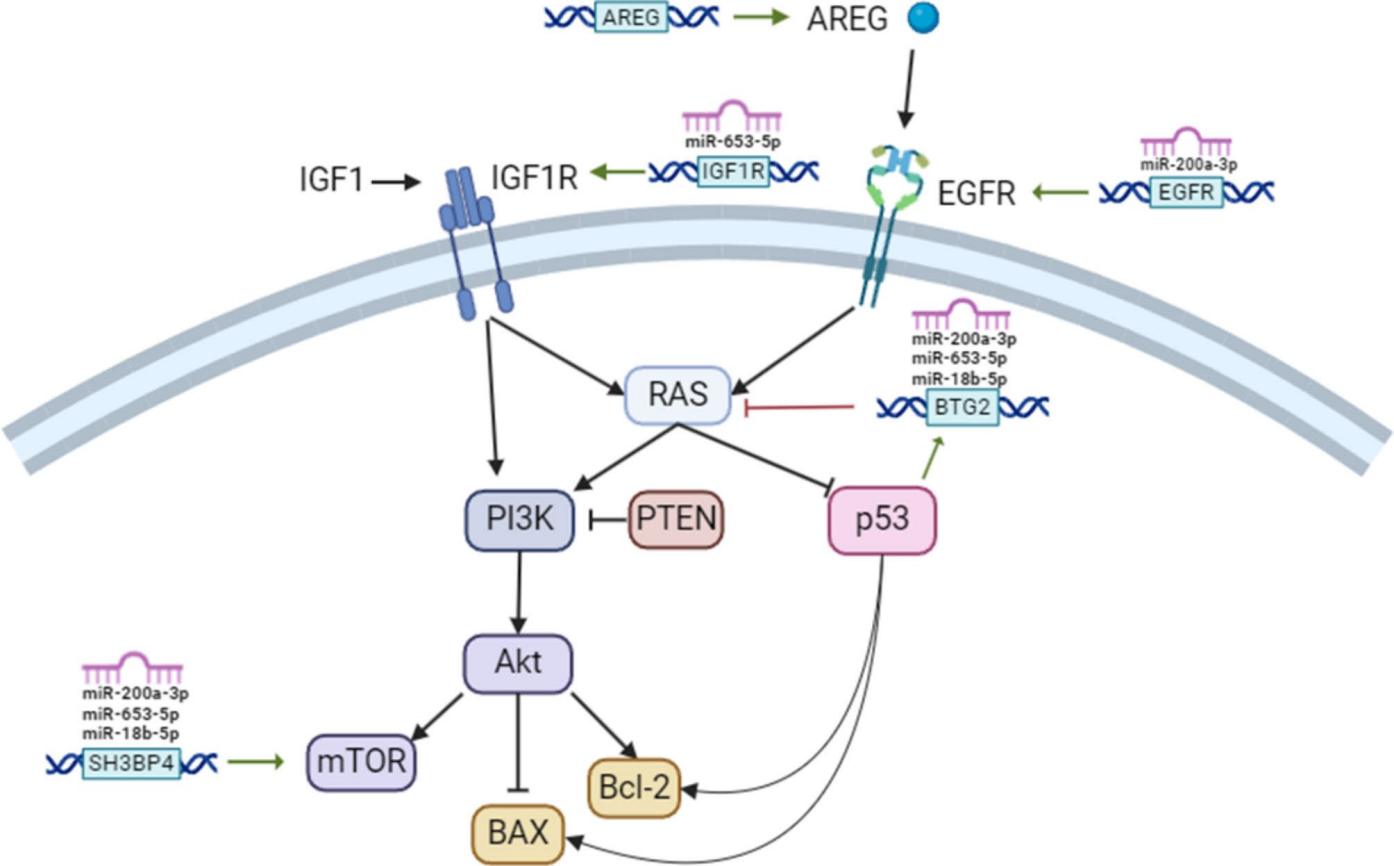
Schematic representation of the mechanisms involving miRNAs regulation in apoptotic and survival cellular pathway.

In summary, this research reveals a complex modulation mechanism, as illustrated in Fig. 9. The exposure to EE2 and DEP, even at subtoxic concentrations, impacts neuronal cells. Firstly, it was observed that both chemicals significantly downregulate key microRNAs—miR-18b-5p, miR-200a-3p, and miR-653-5p. These microRNAs are integral to regulating cellular processes such as proliferation, survival, and apoptosis, and their altered expression disrupts critical pathways associated with neuronal health and function. Secondly, the study highlighted distinct cellular mechanisms through which EE2 and DEP exert their effects. DEP exposure interferes with PTEN activity, leading to increased phosphorylation of Akt but not mTOR, thereby enhancing cell survival through the PI3K/Akt pathway. In contrast, EE2 enhances phosphorylation within the PI3K/Akt/mTOR pathway, promoting pro-survival signals and reducing apoptotic activity. EE2 also impacts p53 activity, further shifting the balance toward anti-apoptotic outcomes. Lastly, the research demonstrated that both EE2 and DEP influence fundamental signaling pathways, including Ras signaling, brain development, and HIF-1 signaling. These disruptions are accompanied by the upregulation of critical genes such as EGFR, IGF1R, BTG2, and SH3BP4, which are known to regulate neuronal proliferation and survival. Despite their overlapping impacts, EE2 and DEP activate these pathways through distinct mechanisms, highlighting their unique but complementary roles in neurotoxicity. The results represent a significant step in unraveling the complex interactions between environmental contaminants and the deregulation of neuronal cell proliferation, highlighting the urgent need for regulatory measures to protect neuronal health and prevent potential long-term neurotoxic effects in exposed populations. By shedding light on these interactions, we aim to foster informed decision-making and targeted interventions to safeguard human health in response to environmental challenges.”

## Materials and methods

### Cell culture

Human neuronal SH-SY5Y cells were purchased from the Lombardy and Emilia-Romagna Experimental Zootechnic Institute (Brescia, Italy) and were regularly grown at 37 °C in a humidified incubator with 5% carbon dioxide (CO_2_). The cultivation media was Dulbecco’s Modified Eagle Medium (DMEM) supplemented with 10% fetal bovine serum (FBS), 2 mM glutamine, 50 U/mL penicillin, and 50 µg/mL streptomycin (Euroclone S.p.A, Pero, Italy).

### EDCs treatment and dose selection

The doses were selected based on the treatment of undifferentiated SH-SY5Y cells with various concentrations of EDCs and the two higher concentrations that were not cytotoxic (0.1 µM and 1 µM) were chosen for further investigations (data not shown). Differentiated SH-SY5Y cells were incubated with EE2 or DEP 0.1 and 1 µM for 48 h in DMEM without phenol red supplemented with 2% FBS at 37 °C in 5% CO_2_. The stock solution of EE2 and DEP were diluted to reach the concentrations needed in complete medium with a maximum of 0.1% dimethyl sulfoxide (DMSO; Merck Life Science S.r.L.) and each experiment included a vehicle group (Vh, DMSO < 0.1%) as the control.

### Neuronal cells differentiation

SH-SY5Y cells were differentiated to mature neurons using retinoic acid. Briefly, 6 × 10^4^ cells/well were seeded in a 6-well plate or 2 × 10^3^ cells/well in a 96-well plate in DMEM without phenol red supplemented with 10% FBS. After 5 h from plating, the medium was replaced with retinoic acid 10 µM (Merck Life Science S.r.L., Milan, Italy) in DMEM without phenol red supplemented with 2% FBS. The same treatment has been changed every 48 h. Finally, after the third and last treatment with retinoic acid, cells were incubated with EDCs for 48 h.

### Determination of neuronal viability

The modulation of neuronal viability in differentiated SH-SY5Y cells was evaluated using the AlamarBlue HSTM test (Thermo Fisher Scientific, Waltham, MA, USA) due to the tendency of these cells to detach more easily. The test is based on the conversion of resazurin to its reduced form, resorufin^57^. After cell differentiation and EDCs treatment, in each well of a 96-well plate was added the 10× cell viability reagent, 10 µL/well, for 1 h at 37 °C in 5% CO_2_. The quantity of resorufin was determined at 570 nm and reference filter at 690 nm, in a multilabel plate reader (GENios, TECAN^®^, Mannedorf, Switzerland). Data are expressed as the mean ± SD of viability percentage relative to cells treated with the Vh (DMSO < 0.1%).

### Pellet collection

Differentiated SH-SY5Y cells contained in four wells in a 6-well plate (exposed to the same EDC or Vh) were detached with trypsin and combined into a single tube. The tubes were centrifuged, the supernatant was removed, and the pellet was resuspended in DPBS. The suspension was then transferred to new tubes and centrifuged again. The supernatant was removed, and the resulting pellet was stored at -80 °C until RNA or protein extraction.

### RNA extraction

Pure link RNA mini kit assay (Thermo Fisher Scientific) was exploited to extract total RNA, as previously described^19^. A lysis buffer containing 1% β-mercaptoethanol was added to the cell pellet, followed by the addition of 70% ethanol and mixing. The solution obtained was loaded in a specific cartridge, washed and finally RNA was eluted with RNase-free water. The quantification was performed by spectrophotometric analysis using NanoDropTM (Thermo Fisher Scientific) and, at the end, RNA was stored at − 80 °C.

### Quantitative real-time PCR (qRT-PCR) analysis of miRNA expression

To assess miRNAs expression levels, RNA was reverse transcribed to cDNA by Mir-X miRNA First-Strand Synthesis and TB Green qRT-PCR assay (Takara, Mountain View, CA, USA) following the manufacturer’s instructions. The cDNA obtained after the thermal cycles was stored at − 20 °C. The expression of miR-18b-5p, miR-200-3p and miR-653-5p was evaluated according to the same protocol and run in a 7900HT Fast PCR system (Applied BiosystemsTM, Thermo Fisher Scientific). miR-U6 was used as internal reference. Three experiments were run for each sample and the 2^−ΔΔCt^ method was performed for the quantitative analysis. The calibrator of the experiment was the Vh. In Table 1 are listed the primer sequences, drawn using the genome browser Ensembl^58^ and the platform for biotechnology research and development Benchling (Benchling [Biology Software] (2022). Retrieved from https://benchling.com).

**Table 1:** Primers sequence for miRNA validation used for qPCR.

miRNA	Primer Sequence (5′-3′)	Utilization
miR-18b-5p	Forward: TAAGGTGCATCTAGTGCAGTTAG	qPCR
	Reverse: mRQ 3’ Primer (TaKaRa)	
miR-200-3p	Forward: TAACACTGTCTGGTAACGATGT	qPCR
	Reverse: mRQ 3’ Primer (TaKaRa)	
miR-653-5p	Forward: GTGTTGAAACAATCTCTACTG	qPCR
	Reverse: mRQ 3’ Primer (TaKaRa)	
miR-U6	Forward: mRQ 5’ Primer (TaKaRa)	qPCR
	Reverse: mRQ 3’ Primer (TaKaRa)	

### Gene expression analysis by RNA reverse transcription and qRT-PCR

Total RNA was reverse transcribed for gene expression analysis and the High-Capacity RNA-to-cDNA kit (Thermo Fisher Scientific) was utilized according to the manufacturer’s instructions. cDNA was stored at − 20 °C and run in a 7900HT Fast PCR system (Applied BiosystemsTM, Thermo Fisher Scientific). qRT-PCR for epidermic growth factor receptor (EGFR), insulin like growth factor 1 receptor (IGF1R), BTG anti-proliferation factor 2 (BTG2), SH3 domain binding protein 4 (SH3BP4) and amphiregulin (AREG) was then performed using PowerUp SYBR Green Master Mix assay (Thermo Fisher Scientific), exploiting GAPDH as endogenous control. Three experiments were run for each sample and the 2^−ΔΔCt^ method was performed for the quantitative analysis. The calibrator of the experiment was the Vh. The primers’ sequences are listed in Table 2.

**Table 2:** Primers sequence for gene expression used for qPCR.

Gene	Primer Sequence (5′-3′)	Utilization
EGFR	Forward: AGTGTGATCCAAGCTGTCCC	qPCR
	Reverse: ACTGCTGGGCACAGATGATT	
IGF1R	Forward: ATGCTCCAAGGATGCACCAT	qPCR
	Reverse: CTCGATGAGCCCCATGAAGT	
BTG2	Forward: GGCACTCACAGAGCACTACA	qPCR
	Reverse: GGGGTCCATCTTGTGGTTGA	
SH3BP4	Forward: AGCTTGTGATGGCCCTACTG	qPCR
	Reverse: GGTCAGGAGCACAAAGTCCT	
AREG	Forward: TCTGGGAAGCGTGAACCATT	qPCR
	Reverse: AGGCATTTCACTCACAGGGG	

### Western blotting

The activation of EGFR, phosphatase and tensin homolog (PTEN), p53, Bax, and Bcl-2 together with the phosphorylation of protein kinase B (Akt) and mammalian target of rapamycin (mTOR) have been assessed by Western blotting method as previously described^19^. Cells were collected and lysated in a complete buffer containing a cocktail of protease/phosphatase inhibitors (100×) and prepared adding leupeptin (2 µg/mL) and PMSF (100 µg/mL) (Merck Life Science S.r.L.). The Bradford assay was employed to determine protein concentration. The samples of protein lysates (30 µg per sample) were added to the Laemmli Sample Buffer 4x (Bio-Rad Laboratories S.r.L., Segrate, Italy) and loaded on a 4–15% TGX polyacrylamide gels (Bio-Rad Laboratories S.r.L.) for 30 min at 200 V, to separate the proteins. Consequently, gels were photoactivated to allow the immediate visualization of proteins and then electroblotted onto 0.45 nm nitrocellulose membranes for 1 h at 100 V. The membranes were then irradiated with UV excitation to visualize the total protein signal and finally incubated at 4 °C overnight with primary antibodies recognizing EGFR, PTEN, p-Akt, p-mTOR, p53, Bax and Bcl-2 (all 1:1000; Cell Signaling Inc., Euroclone S.p.A). The next day, the membranes were incubated with an anti-rabbit or anti-mouse secondary antibody (1:5000; Jackson ImmunoResearch Europe Ltd, Ely, Cambridgeshire, UK) for 1 h at room temperature (RT). Target bands have been detected by enhanced chemiluminescence (ECL reagents, Bio-Rad Laboratories S.r.L.). The same membranes were re-probed with Akt, mTOR (Cell Signaling Inc., Euroclone S.p.A) and β-actin (Merck Life Science S.r.L.) antibodies (all 1:1000) after the stripping protocol. Data were analysed by densitometry, by Quantity One software (Bio-Rad Laboratories S.r.L.). Values were normalized and expressed as the ratio between phosphorylated form and total protein expression, or as the ratio between the protein of interest and the β-actin signal.

### Ras GTPase activation measurement

Ras protein activation has been carried out using the Active Ras Detection kit (Cell Signaling Inc., Euroclone S.p.A.) following the manufacturer’s instructions. Briefly, cell pellet was lysed with 200 µL of complete Lysis Buffer (prepared by adding 2 µL of PMSF (100 mM) to 200 µL of 1X Lysis/Binding/Wash buffer). After centrifugation, the protein concentration in the supernatant was quantified using the Bradford assay.

After the preparation of the spin cup with glutathione resin and GST-Raf1-RBD, it was possible to transfer all the cell lysate (containing at least 500 µg of total protein) in the cup. The reaction mixture was then incubated at 4 °C for 1 h with gentle rocking. The resin was subsequently washed with 400 µL of Lysis Buffer complete (three times) and then 50 µL of reducing sample buffer (prepared combining 1,5 mg of DTT with 50 µL of 2X SDS Sample Buffer, for a final concentration of 200 mM) was added to the resin. After vortexing, the tubes were incubated at RT for 2 min. The eluate obtained after centrifugation was then heated at 95 °C for 5 min to prepare the samples for electrophoresis on 4–15% TGX polyacrylamide gels (Bio-Rad Laboratories S.r.L.), following the previously described Western Blotting protocol.

### In silico target analysis

The network analysis to predict the target genes of the differentially expressed miRNAs and their over-representation analysis were performed using mirWalk; validated interaction data from TargetScan, miRDB, and miRTarBase have been collected^59^. All set of genes targeted by the miRNAs were identified and used to predict the associated pathways. The functional enrichment analysis on input gene list was performed using g: Profiler^60^ that enables to maps genes to known functional information sources and detects statistically significantly enriched terms.

The selected pathways have been chosen based on the hypergeometric tests with p-values ≤ 0.001 and the contribution in the mechanism of the EDCs.

### Statistical analysis

PRISM 9 software (GraphPad Software, La Jolla, CA, USA) was employed to perform data analysis, and the difference between groups was analysed using one-way ANOVA with Dunnett post hoc test. A p-value less than 0.05 was considered statistically significant.

## Electronic supplementary material

Below is the link to the electronic supplementary material.

Supplementary Material 1

## Data Availability

All data supporting the findings of this study are available within the paper and its Supplementary Information.
