# Supplementary material for: Impact of 17-alpha ethinyl estradiol (EE2) and diethyl phthalate (DEP) exposure on microRNAs expression and their target genes in differentiated SH-SY5Y cells
Source: Sci Rep. 2025 Jan 21;15:2722. doi: 10.1038/s41598-025-86911-1 (PMC11751492; doi:10.1038/s41598-025-86911-1)
Supplement: Supplementary file 1 — Supplementary Material 1 [file 41598_2025_86911_MOESM1_ESM.pdf]

Figure 6b and 6e

EGFR

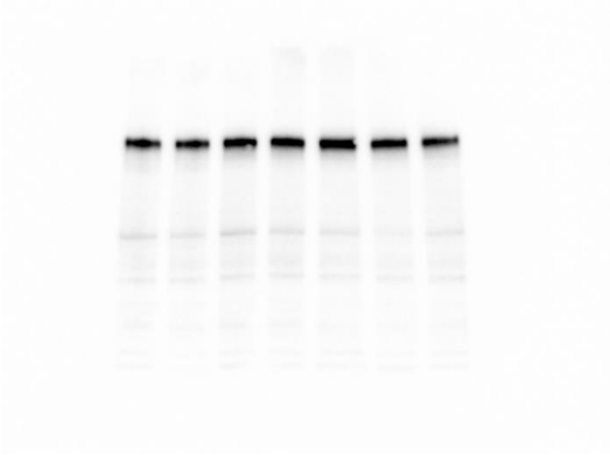

β-actin

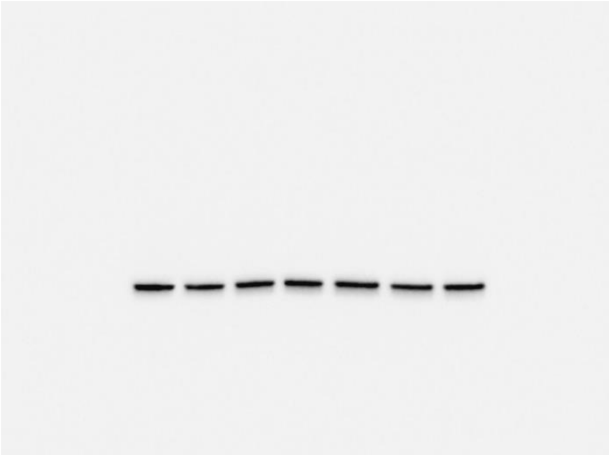

Lane 1: DEP 1 μM
Lane 2: DEP 0.1 μM
Lane 5: EE2 1 μM
Lane 6: EE2 0.1 μM
Lane 7: Vh

Figure 6c

Ras

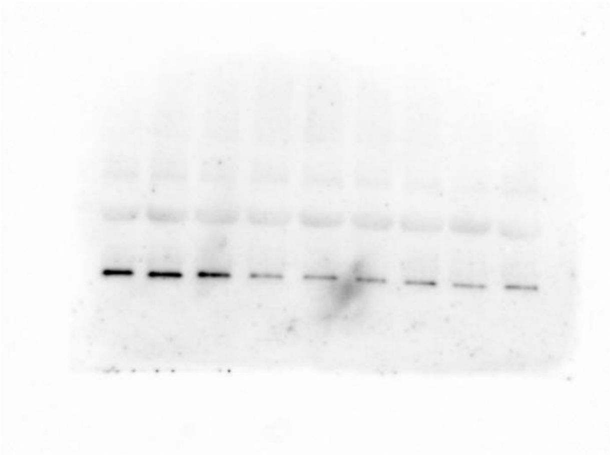

Lane 1: DEP 1 μM
Lane 2: DEP 0.1 μM
Lane 3: Vh

Figure 6g

Ras

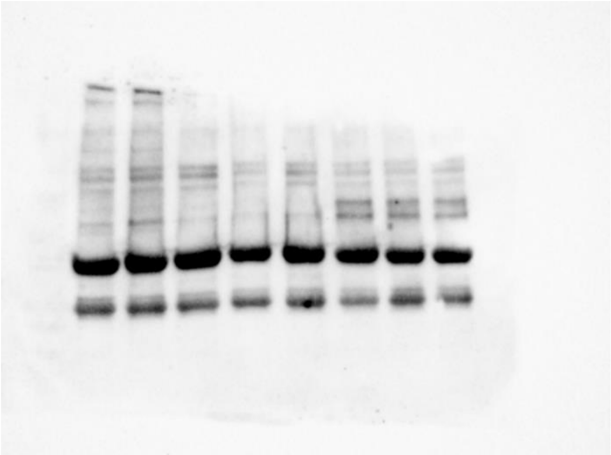

Lane 6: EE2 1 μM
Lane 7: EE2 0.1 μM
Lane 8: Vh

Figure 6d and 6h

p53

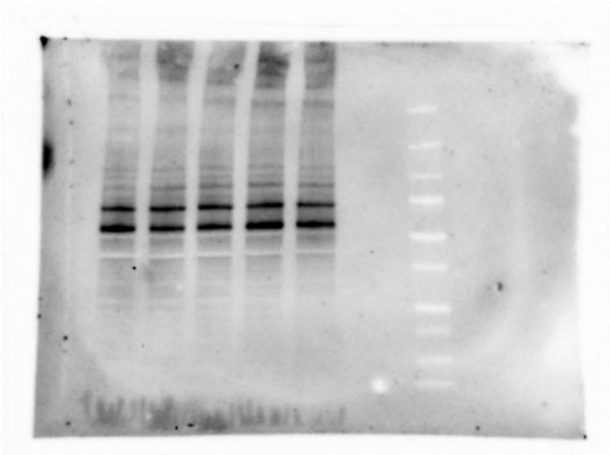

β-actin

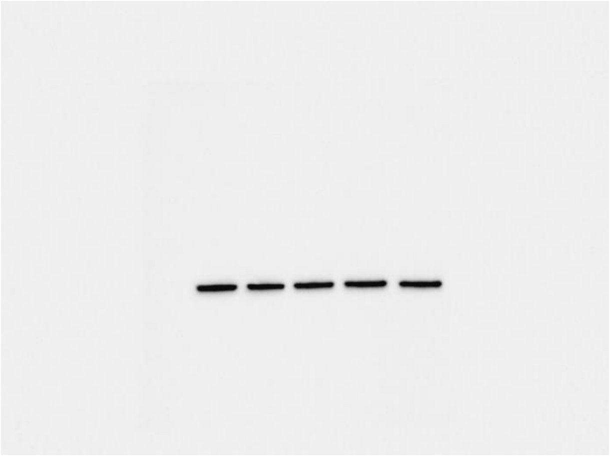

Lane 1: Vh
Lane 2: EE2 0.1 μM
Lane 3: EE2 1 μM
Lane 4: DEP 0.1 μM
Lane 5: DEP 1 μM

Figure 7a and 7d

PTEN

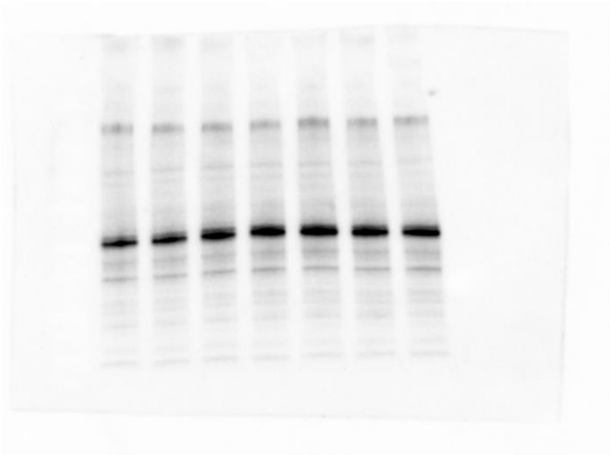

β-actin

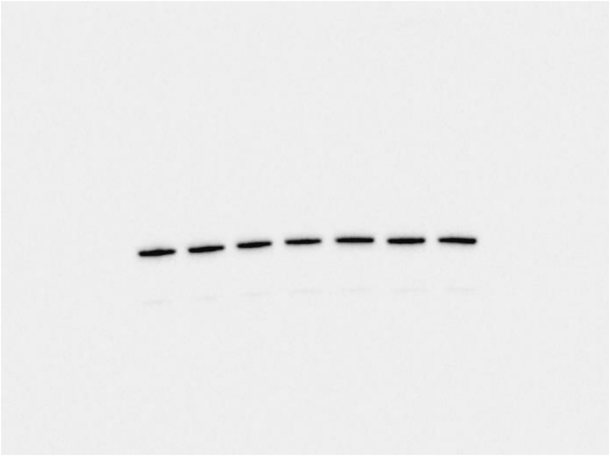

Lane 1: DEP 1 μM
Lane 2: DEP 0.1 μM
Lane 5: EE2 1 μM
Lane 6: EE2 0.1 μM
Lane 7: Vh

Figure 7b

p-Akt

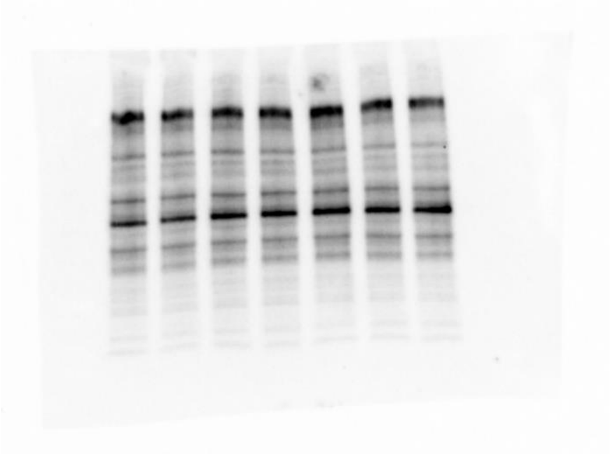

Akt

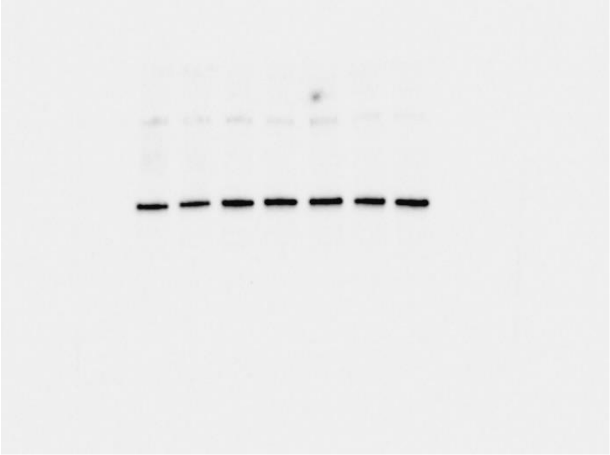

β-actin

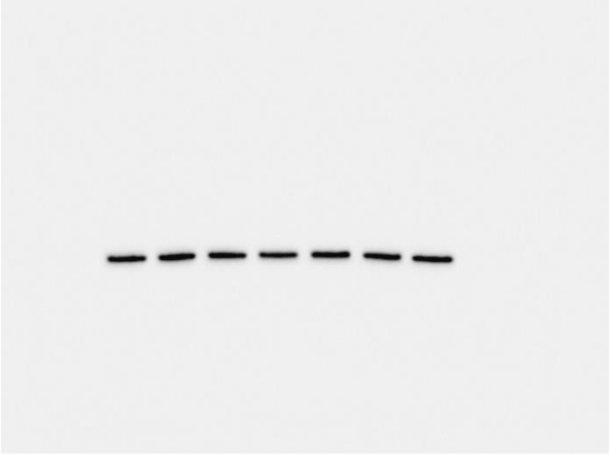

Lane 1: Vh
Lane 6: DEP 0.1 μM
Lane 7: DEP 1 μM

Figure 7e

p-Akt

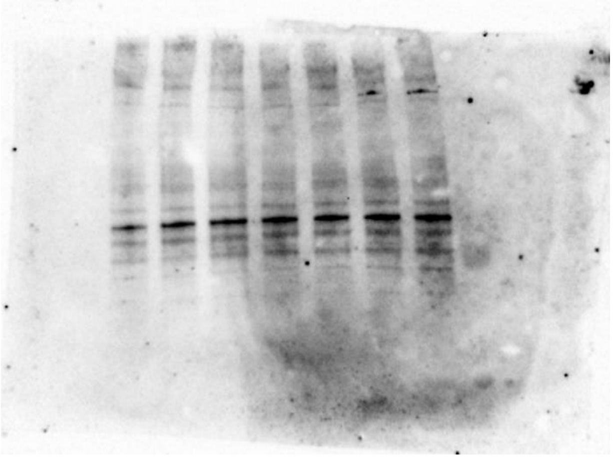

Akt

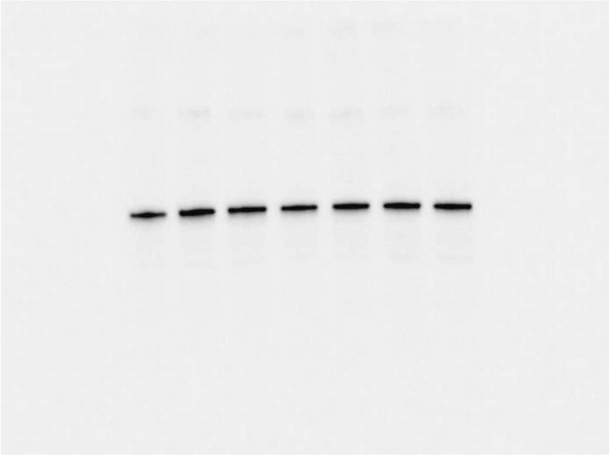

β-actin

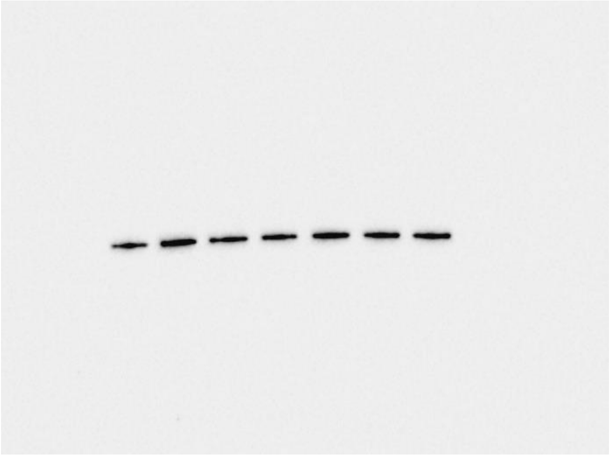

Lane 5: EE2 0.1 μM
Lane 6: EE2 1 μM
Lane 7: Vh

Figure 7c and 7f.

p-mTOR

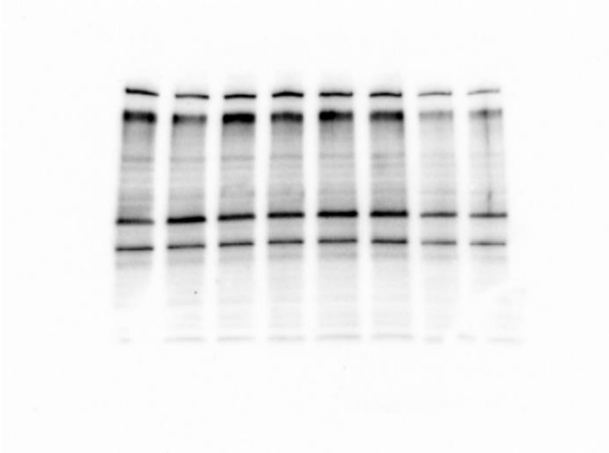

mTOR

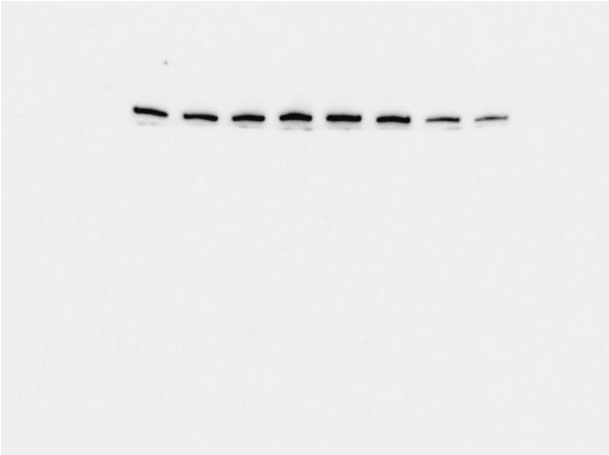

β-actin

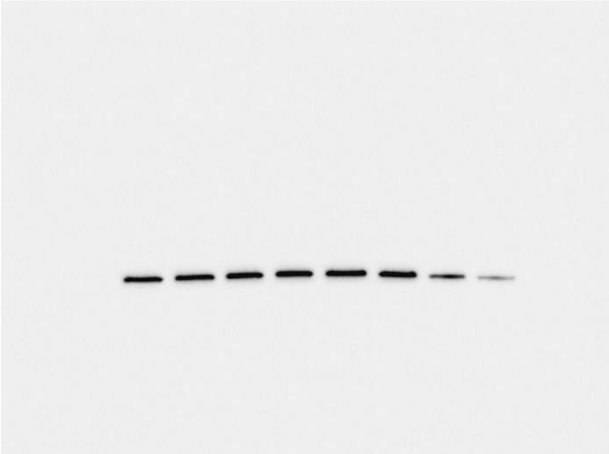

Lane 1: DEP 1 μM
Lane 2: DEP 0.1 μM
Lane 5: EE2 1 μM
Lane 6: EE2 0.1 μM

Lane 7: Vh
Figure 8a

Bax

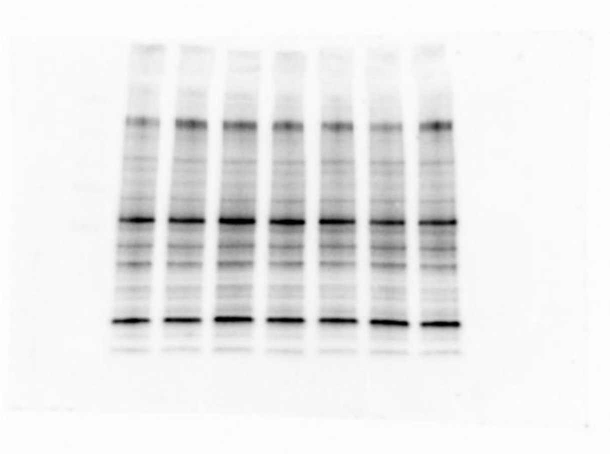

β-actin

Lane 1: DEP 1 μM
Lane 2: DEP 0.1 μM
Lane 7: Vh

Figure 8d
Bax

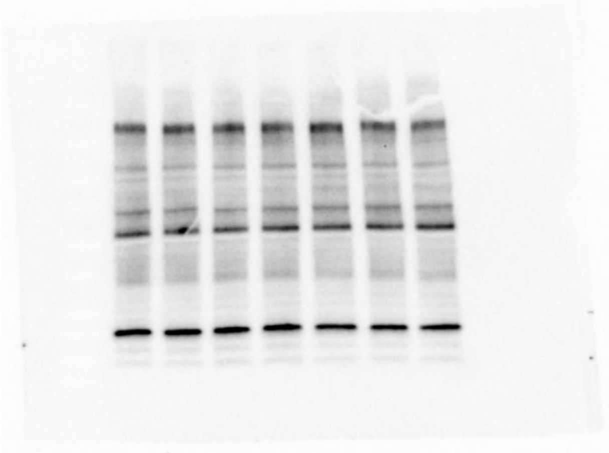

β-actin

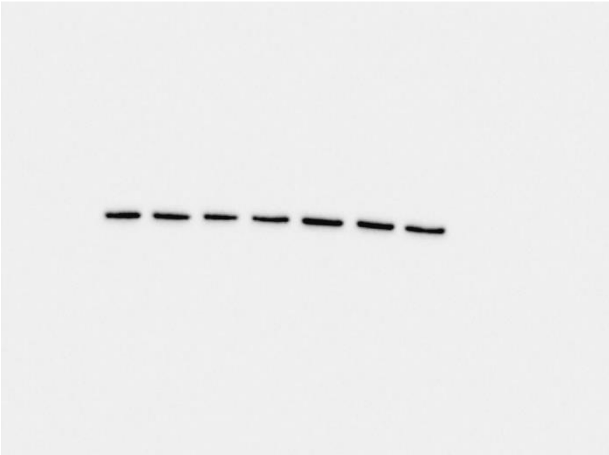

Lane 5: EE2 1 μM
Lane 6: EE2 0.1 μM
Lane 7: Vh

Figure 8b and 8e
Bcl-2

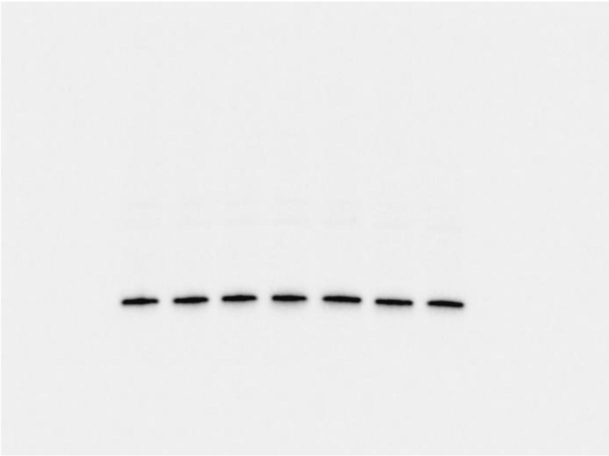

β-actin

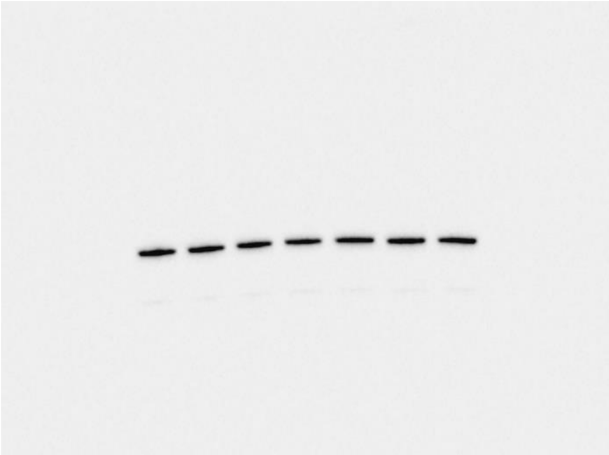

Lane 1: DEP 1 μM
Lane 2: DEP 0.1 μM
Lane 5: EE2 1 μM
Lane 6: EE2 0.1 μM
Lane 7: Vh
